# Supplementary material for: Splice-Junction-Based Mapping of Alternative Isoforms in the Human Proteome
Source: Cell Rep. Author manuscript; Available in PMC 2020 Jan 15. (PMC6961840; doi:10.1016/j.celrep.2019.11.026)
Supplement: 2 [file NIHMS1546469-supplement-2.zip › DF1/PXD010154/Colon/Colon_9_HERC2_WALWSVEAQAR.pdf]

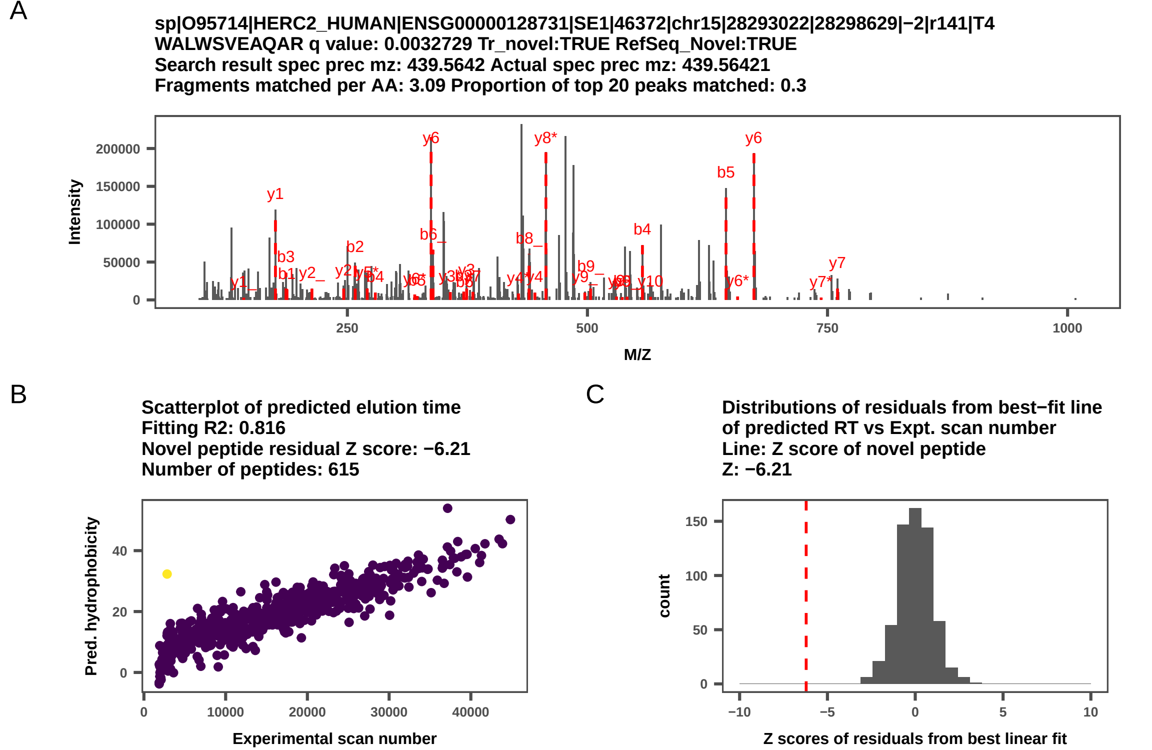
A
sp|O95714|HERC2_HUMAN|ENSG00000128731|SE1|46372|chr15|28293022|28298629|−2|r141|T4
WALWSVEAQAR q value: 0.0032729 Tr_novel:TRUE RefSeq_Novel:TRUE
Search result spec prec mz: 439.5642 Actual spec prec mz: 439.56421
Fragments matched per AA: 3.09 Proportion of top 20 peaks matched: 0.3
Intensity
200000
150000
100000
50000
0
250
500
750
1000
M/Z
y6
y8*
y6
b5
y1
b4
b6_
b8_
b2
b3
b9_
y7
y10
y7*
y6*
B
Scatterplot of predicted elution time
Fitting R2: 0.816
Novel peptide residual Z score: −6.21
Number of peptides: 615
Pred. hydrophobicity
40
20
0
0
10000
20000
30000
40000
Experimental scan number
C
Distributions of residuals from best−fit line
of predicted RT vs Expt. scan number
Line: Z score of novel peptide
Z: −6.21
count
150
100
50
0
−10
−5
0
5
10
Z scores of residuals from best linear fit
